# Deregulation of CREB Signaling Pathway Induced by Chronic Hyperglycemia Downregulates NeuroD Transcription

**DOI:** 10.1371/journal.pone.0034860

**Published:** 2012-04-03

**Authors:** In-Su Cho, Miyoung Jung, Ki-Sun Kwon, Eunpyo Moon, Jang-Hyeon Cho, Kun-Ho Yoon, Ji-Won Kim, Young-Don Lee, Sung-Soo Kim, Haeyoung Suh-Kim

**Affiliations:** 1 Department of Anatomy, Ajou University, Suwon, South Korea; 2 Department of Biological Sciences, Ajou University, Suwon, South Korea; 3 Graduate Neuroscience Program, Ajou University, Suwon, South Korea; 4 BK21, Division of Cell Transformation and Restoration, Ajou University, Suwon, South Korea; 5 Molecular Science and Technology, Ajou University, Suwon, South Korea; 6 Control for Cell Death Regulating Biodrug, Ajou University, Suwon, South Korea; 7 Aging Research Center, Korea Research Institute of Bioscience and Biotechnology (KRIBB), Daejeon, South Korea; 8 Department of Molecular and Cellular Biology, Baylor College of Medicine, Houston, Texas, United States of America; 9 Department of Endocrinology, Catholic University, School of Medicine, Seoul, South Korea; University of British Columbia, Canada

## Abstract

CREB mediates the transcriptional effects of glucose and incretin hormones in insulin-target cells and insulin-producing β-cells. Although the inhibition of CREB activity is known to decrease the β-cell mass, it is still unknown what factors inversely alter the CREB signaling pathway in β-cells. Here, we show that β-cell dysfunctions occurring in chronic hyperglycemia are not caused by simple inhibition of CREB activity but rather by the persistent activation of CREB due to decreases in protein phophatase PP2A. When freshly isolated rat pancreatic islets were chronically exposed to 25 mM (high) glucose, the PP2A activity was reduced with a concomitant increase in active pCREB. Brief challenges with 15 mM glucose or 30 µM forskolin after 2 hour fasting further increased the level of pCREB and consequently induced the persistent expression of ICER. The excessively produced ICER was sufficient to repress the transcription of NeuroD, insulin, and SUR1 genes. In contrast, when islets were grown in 5 mM (low) glucose, CREB was transiently activated in response to glucose or forskolin stimuli. Thus, ICER expression was transient and insufficient to repress those target genes. Importantly, overexpression of PP2A reversed the adverse effects of chronic hyperglycemia and successfully restored the transient activation of CREB and ICER. Conversely, depletion of PP2A with siRNA was sufficient to disrupt the negative feedback regulation of CREB and induce hyperglycemic phenotypes even under low glucose conditions. Our findings suggest that the failure of the negative feedback regulation of CREB is the primary cause for β-cell dysfunctions under conditions of pathogenic hyperglycemia, and PP2A can be a novel target for future therapies aiming to protect β-cells mass in the late transitional phase of non-insulin dependent type 2 diabetes (NIDDM).

## Introduction

Glucose has a critical influence on β-cell functions and mass. Transient increases in glucose concentration within the physiological range promote insulin biosynthesis and secretion. Chronic elevation of glucose level, however, exerts adverse effects on β-cells and leads to decreases in β-cell survival and β-cell-enriched gene expression; the phenomenon is termed “glucotoxicity”. Thus, preservation of β-cell mass against glucotoxicity has become a major point of research in type 2 diabetes. Extensive studies have revealed that chronic hyperglycemic environment imposes various types of stress on β-cells including oxidative stress, endoplasmic reticulum stress, cytokine-induced apoptosis, and hypoxia [1]. However, it is not yet clear how those stress progressively leads to defects in insulin gene synthesis/secretion, and the loss of functional β-cell mass.

Insulin gene expression is regulated by the combined actions of β-cell-specific transcription factors that are also required for the development and survival of pancreatic islet cells. These factors include Pdx-1/IPF-1, Pax4, Pax6, NeuroD/BETA2, Nkx2.2, and MafA [2], [3]. In particular, NeuroD/BETA2, a basic helix-loop-helix transcription factor, directly regulates insulin gene transcription [4], also regulates β-cell-specific genes that are necessary for glucose homeostasis such as sulfonylurea receptor I (SUR1) [5]. NeuroD-deficient mice die at early ages due to severe diabetes [6], or if they survive to adulthood in a different genetic background, β-cells remain immature and lose functional glucose-responsiveness [7]. Transduction of the NeuroD protein can alleviate diabetic symptoms in a type 1 diabetic mouse model [8]. In humans, mutations in NeuroD can predispose individuals to develop maturity onset diabetes of the young (MODY) [9]. Given a critical role of NeuroD in the developing pancreatic islet cells and in mature β-cells, efforts toward functional conservation of the NeuroD gene may effectively treat diabetes mellitus.

cAMP response element binding protein (CREB) is known to play a key role in maintaining glucose homeostasis by mediating the transcriptional effects of glucose and incretin hormones [10]. The functions of CREB have been mostly characterized in association with its cofactor, CRTC2, in the process of fasting-associated gluconeogenesis in insulin-target tissues such as liver and skeletal muscle [11]. By comparison, the roles of CREB in insulin producing β-cells are relatively unknown except that inhibition of CREB in transgenic mice with a dominant negative A-CREB causes severe hyperglycemia due to the loss of β-cell mass [12]. Although those data indicate that CREB is important for preservation of functional β-cell mass, it is still unknown what factors inversely alter the CREB signaling pathway in β-cells.

CREB is activated by phosphorylation at Ser133 in response to increases in intracellular levels of Ca^2+^ or cAMP but deactivated by dephosphorylation of Ser133 by Ser/Thr protein phosphatases, including PP1, PP2A, and PP2B/calcineurin [10], [13]. These phosphatases play diverse roles in various cell types, including β-cells [14]. Clinical and ethnographical studies with Pima Indians also have suggested that defects in PP1 and PP2A are associated with diabetes [15]–[18]. Sato *et al.*, 1998 [19] have showed that okadaic acid, a universal inhibitor of Ser/Thr phosphatases, inhibits insulin secretion by disrupting Ca^2+^ signaling. Depletion of PP2A catalytic subunits using small interfering RNA markedly attenuates glucose-stimulated insulin secretion from pancreatic β-cells [20]. Given that Ser/Thr protein phosphatases have broad substrate specificity, it is predictable that their modulation may affect glucose homeostasis in similar ways. However, mice either lacking or overexpressing PP2B/calcineurin in β-cells ironically display similar diabetic symptoms [21], [22], while PP2A null mice are embryonically lethal [23]. Thus, the precise functions of protein phosphatases are yet to be determined with respect to β-cell functions or diabetes.

CREB signals can be terminated via a negative feedback control by ICER (inducible cAMP early repressor) [24]. ICER mRNA is generated from the CRE-containing intronic P2 promoter of the cAMP response element modulator (CREM) gene, a closely related in structure to CREB, in response to accumulation of active pCREB. ICER proteins dimerize with CREB or CREM activators, and the dimers, in turn, switch off CRE-mediated gene expression including ICER itself. In β-cells, insulin is known as a direct target of ICER [25]. ICER expression is elevated in hyperglycemic rodent islets [26]–[28]. Transgenic mice overexpressing ICER in pancreatic β-cells exhibit severe diabetic symptoms at early ages because of a decrease in β-cell mass [29]. Because insulin deficiency cannot fully ascribe for the loss of β-cell mass at early ages, other factors that regulate differentiation and survival of β-cells may be involved.

In the present study, using freshly isolated rat islet cells as an *ex vivo* system that allows molecular-level studies under physiological and pathophysiological conditions (normoglycemica *versus* hyperglycemia), we show that the decreased level of PP2A levels and the consequent upregulation of ICER is the primary cause for the failure of the negative feedback regulation of CREB under chronic hyperglycemic conditions. We also show that the deregulation of CREB signaling pathway is a key mechanism for silencing of β-cell specific genes such as NeuroD and insulin in the progression of β-cell failure as a complication of diabetes.

## Results

### Chronic exposure to high glucose prolongs ICER expression in hyperglycemic islets

**Figure 1:**
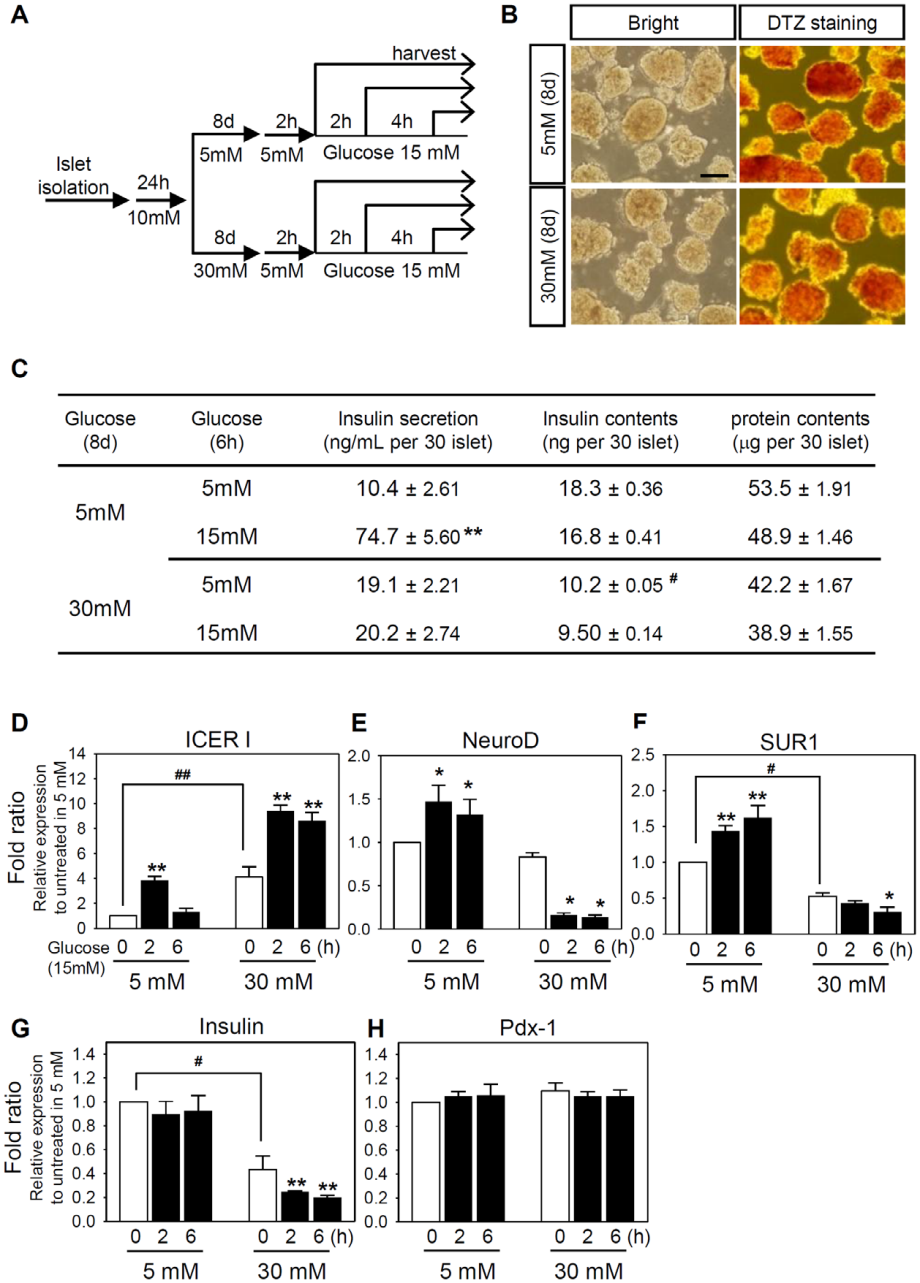
Chronic hyperglycemia alters the β-cell specific gene expression in pancreatic islets. (**A**) Rat islet cells were cultured in the presence of 5 mM or 30 mM glucose for 8 days, and stimulated with 15 mM glucose for 0–6 h after 2 h preconditioning in 5 mM glucose. (**B**) Dithizone staining of islet cells after 8 days of culture. Scale bar: 100 µm. (**C**) In low (5 mM) glucose condition, insulin secretion was normal in response to acute stimulation with 15 mM glucose after 8 day culture whereas insulin secretion was impaired after 8-day exposure to high (30 mM) glucose. Under high glucose conditions, cellular insulin content were significantly reduced, and total proteins were decreased slightly. (**D∼H**) Real-time PCR was carried out using SYBR green to quantitate the mRNA levels of indicated genes in diverse conditions shown in **A**. Relative mRNA levels were estimated from of Ct values summarized in Supporting Table S1 using 2^−ΔΔCt^ method. The data from three independent experiments are presented as average fold ratios (means ± S.E.) of relative mRNA expression compare with the 5 mM glucose-cultured islets before glucose stimulation. Simultaneous decreases in the mRNA levels and the intracellular insulin content correlated well. Significant effects of 15 mM glucose (*, *P*<0.05; **, *P*<0.01) or 8-day incubation in 30 mM glucose (*^#^*, *P*<0.05) are marked. Similar results were obtained with traditional, semi-quantitative methods (Supporting Figure S1).

To establish a cellular glucotoxicity model, rat pancreatic islets were freshly isolated and grown in RPMI medium containing 30 mM (high level) or 5 mM (low level) glucose for 8 days (Figure 1A). To validate 8-day cultivation in 30 mM glucose could mimic chronic hyperglycemia *in vivo*, we tested insulin secretion capability. In low glucose conditions, the islets retained the original aggregated morphology (Figure 1B) as well as the capability to secrete insulin in response to acute challenges with 15 mM glucose following 2 h-fasting in 5 mM glucose (Figure 1C). In contrast, 8-day cultivation in the presence of 30 mM glucose blunted glucose-stimulated insulin secretion. Concomitantly, the total insulin content in the islets was also reduced after 8-day cultivation in 30 mM glucose (Figure 1C). Decrease in insulin secretion and insulin content are the indication of glucotoxicity found in chronic hyperglycemia [1]. Hence, the experimental condition described in Figure 1A provides a proper cellular glucotoxicity model for the analysis of impaired β-cell specific gene expression. The total RNA was isolated from each of 30 islets and the expression levels of β-cell specific genes were quantitated using SYBR green real-time PCR (Supporting Table S1). When islets were cultivated in the presence of 5 mM glucose, acute stimulus with 15 mM glucose slightly increased the mRNA levels of NeuroD and SUR1 within 6 hours whereas the insulin transcription was not altered (Figure 1E–1G). Importantly, ICER expression was transiently increased by 3.8-fold at 2 h but fell to the basal level at 6 h (Figure 1D). In contrast, when islets were cultivated in the presence of 30 mM glucose, the insulin transcription was reduced by 58% (Figure 1G). Concomitantly, the mRNA levels of NeuroD, SUR1, and insulin were reduced by 86%, 72%, and 81% after 6 hour challenge with 15 mM glucose, respectively (Figure 1E–1G). Importantly, the mRNA level of ICER was persistently elevated in 30 mM glucose and further increased by 15 mM glucose stimulus up to 9.3 folds for 6 hours (Figure 1D). Meanwhile, the mRNA level of Pdx-1 was not altered in any instance (Figure 1H), consistent with the notion that the regulation of Pdx-1 mainly takes place at the post-transcriptional level [30]–[32]. The results also suggest that the reduced intracellular insulin content in chronic hyperglycemia (Figure 1C) are partially due to the decrease in insulin gene transcription (Figure 1G). Traditional, semi-quantitative RT-PCR data performed with HIT cells were also similar to those obtained with SYBR green real-time PCR (Supporting Figure S1). Taken together, these results suggest that chronic exposure to hyperglycemia prolongs the ICER induction. The inverse correlation of ICER with NeuroD, SUR1 and insulin also suggest that these genes may be directly or indirectly regulated by ICER.

### Excessive activation of CREB prolongs ICER induction

**Figure 2 pone-0034860-g002:**
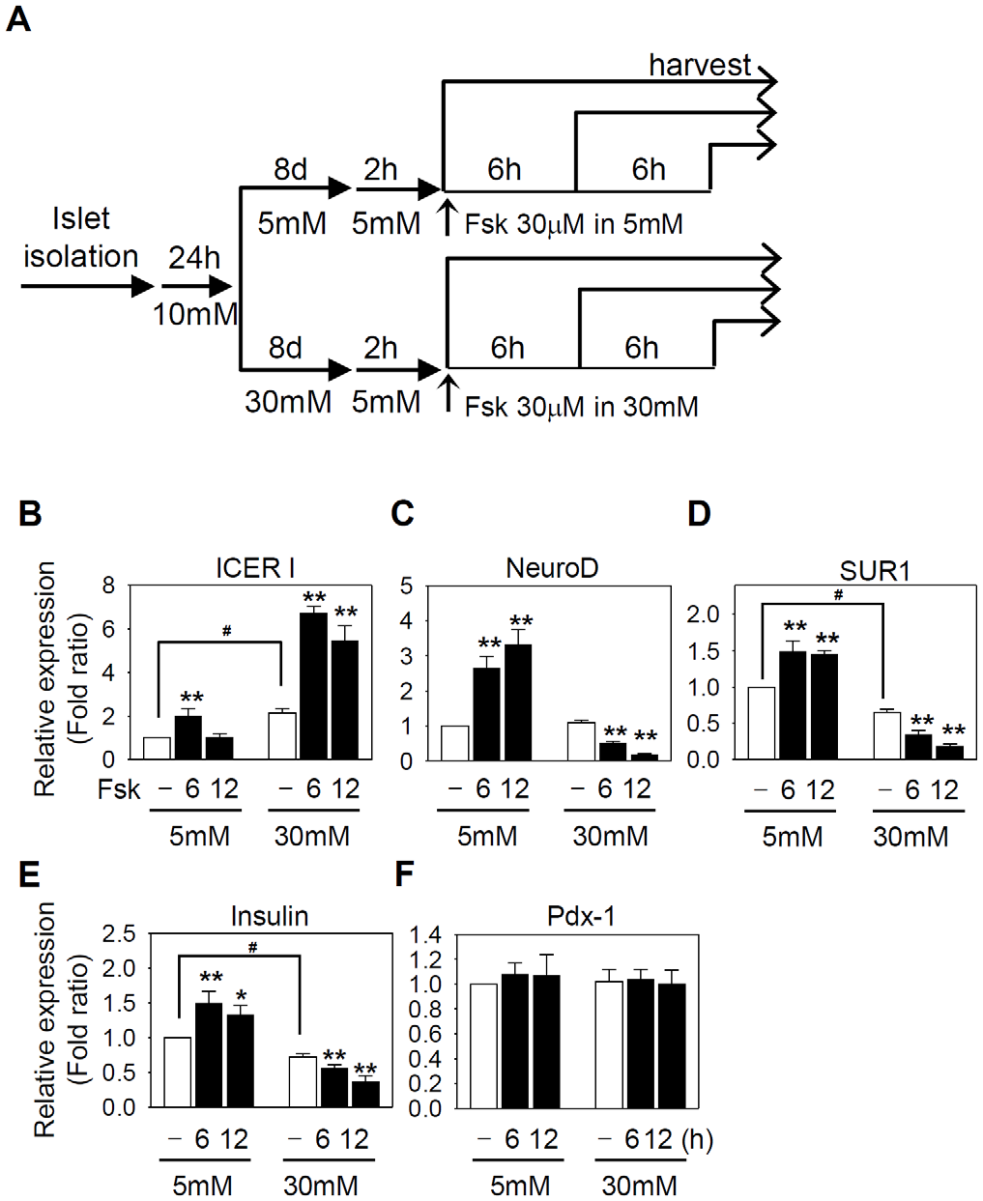
Chronic hyperglycemia alters the responsiveness to cAMP in pancreatic islets. (**A**) Rat islet cells were cultured in the presence of 5 mM or 30 mM glucose for 8 days and stimulated with 30 µM forskolin for 0–12 h after 2 h preconditioning in 5 mM glucose. (**B∼F**) Real-time PCR was carried out using SYBR green to quantitate the mRNA levels of indicated genes in diverse conditions shown in **A**. Relative mRNA levels were estimated from Ct values summarized in Supporting Table S1 using 2^−ΔΔCt^ method. The data from three independent experiments are presented as average fold ratios of relative mRNA expression compare with the 5 mM glucose-cultured islets before forskolin treatment in 5 mM glucose-cultured islets. Significant effects of forskolin (*, *P*<0.05; **, *P*<0.01) or 8-day incubation in 30 mM glucose (*^#^*, *P*<0.05) are marked.

Since ICER is known to be induced by excessive activation of the cAMP-CREB pathway [24], chronic hyperglycemia may also alter the β-cell specific gene expression in response to hormones that increase the intracellular cAMP levels. To mimic hormonal effects, we added 30 µM forskolin, an activator of adenylyl cyclase, to islet cultures that were maintained in 5 mM or 30 mM glucose for 8 days (Figure 2A). The responsiveness to cAMP was maintained in islet cells cultured in low glucose conditions for 8 days, thus the levels of NeuroD, SUR1, and insulin mRNA were increased for 6–12 h after forskolin treatment (Figure 2C–2E). Importantly, ICER was transiently induced only for 6 h and the ICER level dropped to the basal level at 12 h (Figure 2B). In contrast, after 8-day culture in high glucose conditions, an acute treatment with forskolin increased the ICER transcription for an extended period of time up to 12 h. Under the same condition, the mRNA levels of NeuroD, SUR1, and insulin were reduced by forskolin (Figure 2C–2E). As expected, the transcription of Pdx-1 was not altered (Figure 2F). The results suggest that the impaired gene expression of β-cell specific genes in response to glucose or forskolin (or cAMP) after chronic exposure to hyperglycemia may involve common signaling pathways.

**Figure 3:**
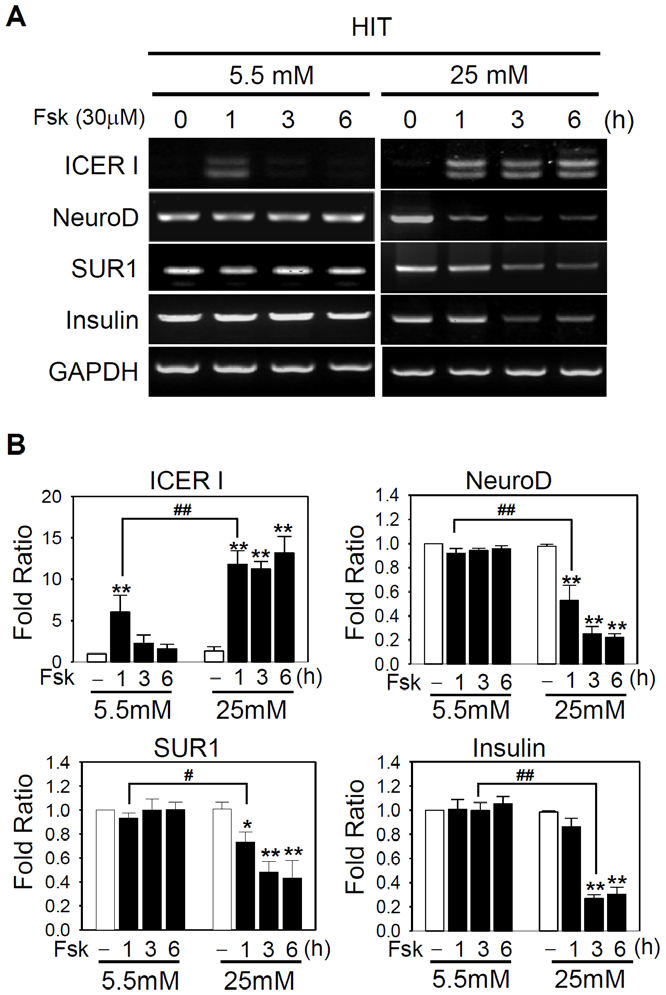
cAMP exerts similar effects in HIT cells after chronic exposure to hyperglycemia. (**A**) HIT cells were grown in 5.5 mM (left panels) or 25 mM glucose (right panels) and treated with 30 µM forskolin for the indicated time. Semi-quantitative RT-PCR analysis revealed a transient (in 5.5 mM glucose) or a persistent (in 25 mM glucose) induction of two isoforms of ICER. (**B**) The mRNA level of each gene was normalized to that of GAPDH. Data from three independent experiments are presented as fold ratios with respect to the value obtained with 5.5 mM glucose in the absence of forskolin. Significant effects of forskolin (*, *P*<0.05; **, *P*<0.01) or chronic 25 mM glucose (*^#^*, *P*<0.05; ^##^, *P*<0.01) are marked.

To understand the molecular mechanisms underlying the distinctive responsiveness to glucose or hormonal stimuli after long-term culture in high and low glucose, we sought for another *in vitro* culture system that could recapitulate the long-term effects of glucose as found in pancreatic islet cells while allowing us to manipulate gene expression. HIT insulinoma cells were grown in DMEM containing 5.5 mM (low) and 25 mM (high) glucose. As traditional, semi-quantitative RT-PCR and real-time PCR with SYBR green yielded qualitatively similar results in islet cells (compare Figures 1 and 2 with Supporting Figures S1 and S2), traditional RT-PCR analysis was carried out to investigate the impaired gene expression. In HIT cells, the responsiveness to cAMP was impaired as found in rat islet cells grown in high glucose (Figure 2 and 3). Thus, forskolin prolonged the ICER induction for longer than 6 h and concomitantly suppressed the level of NeuroD, SUR1, and insulin transcription by 77%, 54%, and 72%, respectively (Figure 3). In contrast, in HIT cells grown in the presence of 5.5 mM (low) glucose, ICER was transiently induced only for 1 h after forskolin treatment but immediately fell to the basal level thereafter. Meanwhile, the expression of NeuroD, insulin, or SUR1 was not affected. The data consistently suggest that persistent induction of ICER is the primary cause for the repression of NeuroD, SUR1, and insulin. The results also indicate that HIT cells can serve as a physiologically relevant *in vitro* system for analyzing the effects of chronic hyperglycemia on ICER-mediated repression of β-cell-specific genes. As NeuroD is a known transcriptional regulator of SUR1 and insulin, we hypothesized that NeuroD might be a direct target of ICER.

### NeuroD is a target gene of ICER

**Figure 4:**
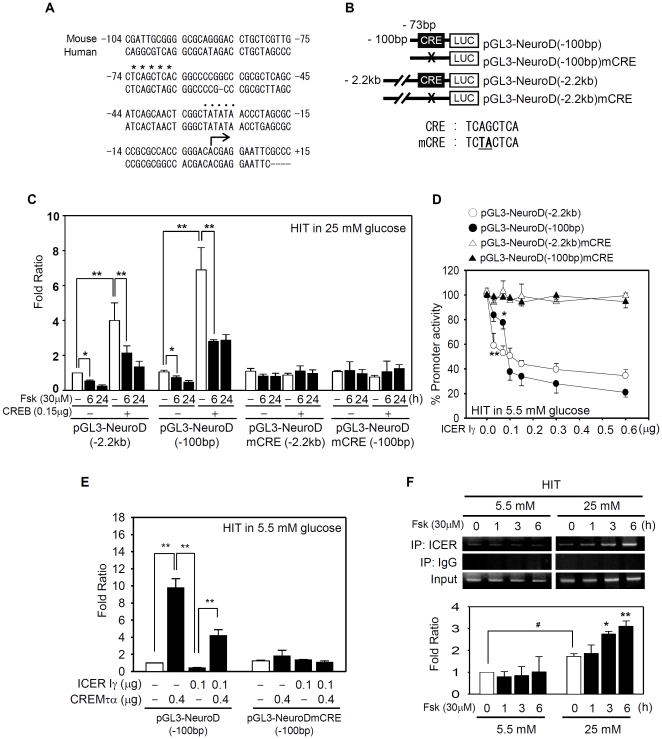
ICER binds to a novel CRE sequence in the proximal NeuroD promoter. (**A**) Comparison of the proximal promoters of mouse (gi:3641530) and human (gi:7416051) NeuroD. The mouse TATA box (••••) at −30 bp and a potential CRE site (****) at −73 bp from the transcription initiation site (the arrow) are conserved. (**B**) A schematic model of reporter genes with −100 bp or −2.2 kb fragment of NeuroD promoter. Sequences for a putative wild type CRE and mutated (mCRE) are shown. (**C**) Reporter genes with the wild type CRE or mCRE (0.4 µg) were cotransfected with the CREB expression vector (0.15 µg) into HIT cells grown in 25 mM glucose. Forskolin (30 µM) was added for the indicated time before harvesting. The average luciferase activity from three independent experiments was shown as the fold ratio with respect to the basal activity of the reporter gene without CREB overexpression and forskolin. (*, *P*<0.05; **, *P*<0.01). (**D**) ICER Iγ directly repressed in a dose dependent manner the NeuroD reporter genes (0.4 µg) only with the wild type CRE but not with a mCRE in HIT cells grown in 5.5 mM glucose. (**E**) HIT cells grown in 5.5 mM glucose were cotransfected with NeuroD reporter genes (0.4 µg) and expression vectors for CREM τα (0.4 µg) and/or ICER Iγ (0.1 µg). The relative luciferase activity from three independent experiments is presented as the fold ratio with respect to the value of indicated conditions. (**F**) ChIP assays were performed to detect direct binding of endogenous ICER to the NeuroD CRE sequence at −73 bp using HIT cells grown in 5.5 mM or 25 mM glucose after stimulation with 30 µM forskolin for the indicated time. Long-term culture in 25 mM glucose enhanced the binding of ICER to CRE (right panels). The absence of immunoprecipitated CRE with a nonspecific IgG verified the specificity of the assay. Results from three independent ChIP assays were semi-quantitatively measured and presented as fold ratios with respect to the value obtained without forskolin in 5.5 mM glucose. Significant effects of forskolin (*, *P*<0.05; **, *P*<0.01) or chronic high glucose (*^#^*, *P*<0.05) are marked.

The mouse *NeuroD* gene [33] contains a TATA box in the proximal promoter and two putative canonical CRE-like sequences (TCAGCT^C^/_A_^A^/_G_) at −1001 bp and −73 bp, with reference to the transcription initiation site (Figure 4A). Only the CRE-like sequence at −73 bp is highly conserved among humans, primates, and rodents (Supporting Table S2), suggesting the retention of functional significance throughout evolution.

To determine whether the CRE-like sequence at −73 bp functions as a binding site for ICER, we performed assays with reporter genes driven by the full length, pGL3-NeuroD(−2.2 kb), and minimum promoter, pGL3-NeuroD(−100 bp) (Figure 4B). The 2.2 kb fragment upstream from the transcriptional startpoint is known to be sufficient to direct β-cell-specific expression of NeuroD [34], whereas the 100 bp fragment contains minimal promoter with TATA box and a putative CRE sequence. Overexpression of CREB enhanced the promoter activities of pGL3-NeuroD(−2.2 kb) and pGL3-NeuroD(−100 bp) by 4.1∼6.9-fold in HIT cells (Figure 4C). Importantly, both the basal and CREB-stimulated activity of reporter genes with wild type CRE were downregulated by forskolin treatment (Figure 4C). Overexpression of ICER Iγ repressed the both reporter genes in a dose-dependent manner (Figure 4D) whereas overexpression of CREMτα, a constitutively active form of CREM that has a higher affinity for CRE sequences than CREB [25], [35], attenuated the negative effects of ICER by competing with ICER for binding to the CRE sequence (Figure 4E). In any cases, a mutation of the CRE sequence (mCRE) abolished the effects of CREB, CREMτα, ICER Iγ, or forskolin, verifying the idea that the TCAGCTCA sequence at −73 bp is a functional CRE. Direct binding of ICER to the CRE at the −73 bp position was clearly proven by chromatin immunoprecipitation (ChIP) assays performed with an anti-ICER antibody (Figure 4F). Under low glucose conditions, the amount of the ICER-specific product was minor and not increased by forskolin. In contrast, the basal intensity of ChIP product was slightly elevated under high glucose conditions and further increased by 2.3-fold at 6 h after the forskolin treatment. These data collectively suggest that chronic exposure to supraphysiologic glucose concentrations destroys the negative feedback regulation of the CREB-ICER pathway and the excessively produced ICER proteins silence the NeuroD promoter by binding to the CRE.

### Deregulation of CREB signaling by reduced PP2A in chronic hyperglycemia

**Figure 5:**
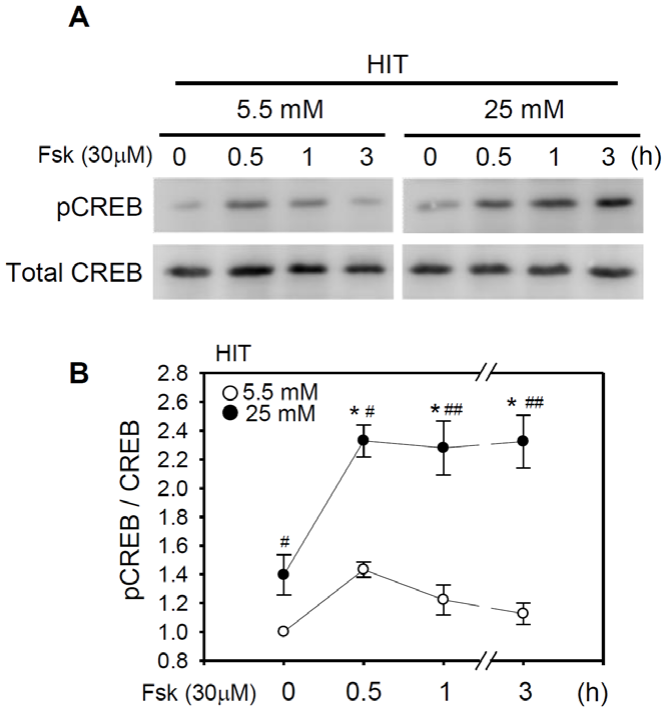
Chronic hyperglycemia persistently activates the basal and forskolin-stimulated pCREB. (**A**) HIT cells were incubated in the presence of 30 µM forskolin for the indicated time before harvesting. Western blot analysis was carried out with an antibody specific for CREB phosphorylated at Ser133 (pCREB). The membrane was re-probed with an anti-CREB antibody to determine the total amount of CREB. (**B**) Levels of pCREB were normalized to that of total CREB. Data from three independent western assays are presented as the fold ratios with respect to the value of HIT cells grown in 5.5 mM glucose in the absence of forskolin. Significant effects of forskolin (*, *P*<0.05; **, *P*<0.01) or chronic high glucose (*^#^*, *P*<0.05) are marked.

ICER expression can be terminated by deactivation of pCREB which otherwise binds to a CRE site in the ICER promoter, and by autologous negative feedback by its own product [24]. Thus, persistent induction of ICER in chronic hyperglycemia suggests that pCREB deactivation does not occur and consequently the pCREB level is constantly elevated. Indeed, western blot analysis indicated that pCREB expression under low glucose conditions was minimal and transiently increased by 1.4-fold only for 30 min after forskolin treatment (Figure 5). By comparison, the basal pCREB level was elevated by 1.4-fold under high glucose conditions, and further increased by forskolin. The elevation of pCREB level longer than 3 h was sufficient to support the persistent ICER induction for an extended period of time.

**Figure 6:**
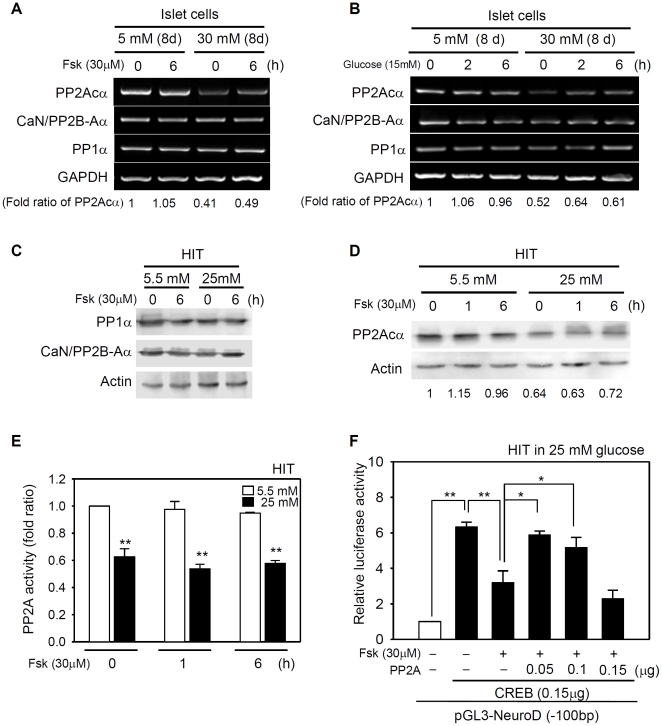
Chronic hyperglycemia reduces the PP2A level. (**A, B**) Rat islet cells grown in 5 mM or 30 mM glucose were treated with 30 µM forskolin for 6 h (the same conditions as shown in Figure 2A). Alternatively rat islet cells grown in 5 mM or 30 mM glucose for 8 days were stimulated with 15 mM glucose for the indicated times (the same conditions as shown in Figure 1A). RT-PCR analysis was employed to determine the relative PP1α, PP2A Cα, and calcineurin/PP2B-Aα mRNA levels. The average values of PP2A Cα from three independent experiments are presented in the bottom of the gel as the relative ratios to the basal expression value in 5 mM glucose. (**C, D**) Western analysis performed with HIT cells grown in 5.5 mM or 25 mM glucose. PP1, PP2A, and PP2B level was normalized to that of actin. The average values from three independent experiments are presented in each lane as the relative ratio to the basal expression level in 5.5 mM glucose. (**E**) HIT cell lysates were immunoprecipitated with anti-PP2A antibody and subject to a standard phosphatase assay with a synthetic peptide. Data from three independent experiments are presented as fold ratios with respect to the value obtained in 5.5 mM glucose (**, *P*<0.01). (**F**) HIT cells grown in 25 mM glucose were cotransfected with 0.4 µg NeuroD reporter gene and 0.15 µg CREB expression vector, together with indicated amount of the PP2A Cα expression vector. Forskolin was added to a final concentration of 30 µM for 6 hour. The relative luciferase activity from three independent experiments is presented as the fold ratio with respect to the value of reporter gene alone (*, *P*<0.05; **, *P*<0.01).

To estimate the deactivation pathway of pCREB, we firstly identified Ser/Thr protein phosphatases responsible for dephosphorylation of CREB Ser133 by RT-PCR analysis of protein phosphatases that were previously found in β-cells [14]. Among the catalytic subunit of PP1, PP2A, and PP2B/calcineurin, we found that only the PP2A expression was downregulated more than 50% in pancreatic islets after chronic exposure to high glucose (Figure 6A and 6B). Under the same condition, PP1 or PP2B/calcineurin was not affected at all. Similarly, the protein levels of PP1α and calcineurin/PP2Bα were not reduced in HIT cells (Figure 6C) under the condition that the PP2A protein level was decreased (Figure 6D). The data indicate that PP2A is the major protein phosphatase that is downregulated by chronic hyperglycemia in pancreatic islet cells. Consistently, the enzyme activity of PP2A was also decreased after chronic exposure to high glucose (Figure 6E), being consistent with the persistent elevation of pCREB or ICER expression. Thus, moderate overexpression of the PP2A Cα (0.05–0.1 µg) negated the inhibitory effects of forskolin and restored the CREB-mediated induction of the NeuroD promoter to the value in the absence of forskolin (Figure 6F). By comparison, excessive expression of PP2A Cα (0.15 µg) lowered the CREB-mediated induction of the NeuroD probably by abrogating even the beneficial effect of pCREB (Figure 6F). When HIT cells were grown in 5.5 mM glucose, acute challenges with 15 mM glucose or 30 µM forskolin did not significantly alter the levels of PP2A Cα mRNA, protein, and enzyme activity for 6 h. The results collectively suggest that the PP2A activity predisposed by long-term culture (low *versus* high glucose conditions) is a determinant for the basal pCREB level and the subsequent duration of ICER induction (transient *versus* persistent) in response to acute stimulation with glucose or forskolin.

### Reduced activity of PP2A is the primary cause of impaired gene expression

**Figure 7:**
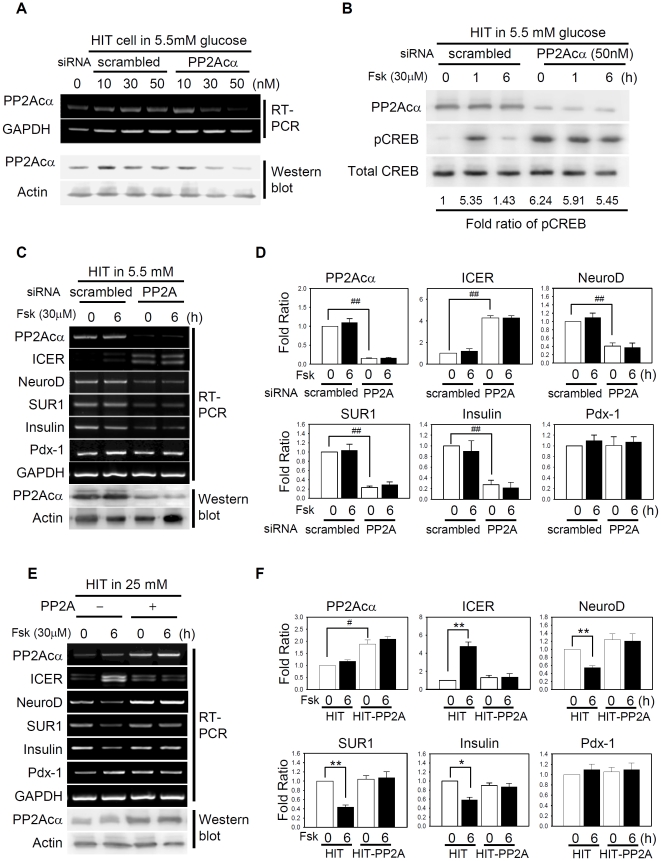
Reduced activity of PP2A is the primary cause of impaired gene expression. (**A**) HIT cells were transfected with the indicated amounts of PP2A Cα siRNA for 48 h. RNA was harvested and subjected to RT-PCR analysis or proteins subject to western analysis. Scrambled RNA was employed to validate the specificity of PP2A Cα-specific siRNA. (**B**) HIT cells grown in 5.5 mM glucose were transfected with 50 nM siRNA specific for PP2A Cα or with scrambled sequences. Prior to harvesting, 30 µM forskolin was added to the culture for 1–6 h. Western analysis was performed with anti-phospho CREB (pCREB Ser133) or PP2A Cα antibody, followed by antibody against total CREB. (**C**) RT-PCR analysis with HIT cells grown in 5.5 mM glucose indicated that PP2A Cα-specific siRNA altered the basal and forskolin-induced gene expression of ICER, NeuroD, SUR1, and insulin as shown in hyperglycemic conditions. (**E**) RT-PCR analysis with HIT cells stably overexpressing PP2A Cα. Overexpression of PP2A in HIT cells grown in 25 mM glucose restored the ICER, NeuroD, SUR1, and insulin to the values obtained in normoglycemic (5.5 mM) condition. (**D, F**) Data from three independent experiments shown in **C, E** are presented as fold ratios with respect to the value of scrambled siRNA or mock-transfected HIT cells (*^#^*, *P*<0.05; *^##^*, *P*<0.05), and to the value of without forskolin (*, *P*<0.05; **, *P*<0.01).

We hypothesized that simple depletion of PP2A under low glucose conditions would mimic hyperglycemic condition and evoke responses of chronic hyperglycemia by forskolin treatment. The siRNA was designed to target PP2A Cα that is highly conserved among species [36] including hamster-derived HIT cells (Supporting Figure S3). Depletion of PP2A Cα was verified by RT-PCR and western analysis (Figure 7A). In HIT cells transfected with PP2A Cα-specific siRNA, pCREB level was persistently enhanced in the presence of 5.5 mM glucose regardless of forskolin treatment (Figure 7B). Concomitantly, ICER was persistently expressed while expressions of NeuroD, SUR1, and insulin were repressed (Figure 7C and 7D). In contrast, stable expression of PP2A Cα prevented the excessive production of ICER and thus, the expression of NeuroD, SUR1, and insulin remained unaffected by forskolin treatment even after long-term culture in 25 mM glucose (Figure 7E and 7F). The results collectively indicate that PP2A is an important player that determines the basal pCREB level and the duration of ICER induction in response to various glucose concentrations, consequently influencing the expression of β-cell-specific genes.

## Discussion

Over the years, knowledge on the mechanisms underlying chronic hyperglycemia-induced glucotoxicity has expanded rapidly, and several candidate components possibly associated with pathogenesis in pancreatic β-cells have been identified. Here, we describe a novel function of PP2A; the phosphatase switches the stimulatory signals of glucose or cAMP to a repressive mode during the pathogenic progress culminating in β-cell dysfunction and failure.

**Figure 8:**
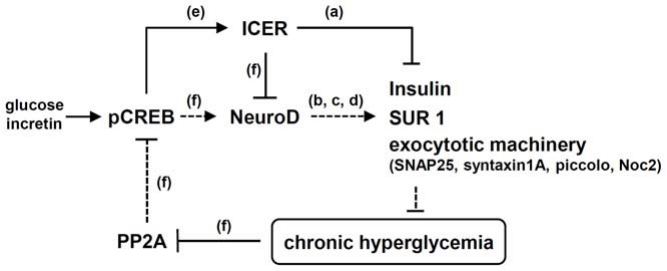
ICER-mediated NeuroD repression aggravates vicious cycle of chronic hyperglycemia. In normoglycemia, PP2A keeps the level of CREB below the physiological threshold and transient activation of CREB in response to glucose and incretin is not sufficient to induce persistent expression of ICER. In chronic hyperglycemia, CREB is constantly activated due to the decreased PP2A level. Upon glucose stimulation or hormonal cues, CREB is further activated for an extended period of time, leading to prolonged ICER induction. Consequently, excessively produced ICER proteins repress the NeuroD expression and the NeuroD's target genes including insulin, SUR1, and components of the exocytotic machinery. The hyperglycemic condition is progressively aggravated through this vicious negative cycle of insulin depletion, and ultimately progressed to β-cell failure. (a) Inada *et al.*, 1999 [25]; Abderrahmani *et al.*, 2006 [28]; (b) Naya *et al.*, 1995 [4]; (c) Kim *et al.*, 2002 [5]; (d) Ishizuka *et al.*, 2007 [43], Gu *et al.*, 2010 [7]; (e) Sassone-Corsi, 1998 [24]; and (f) this study. The active pathways in chronic hyperglycemia are presented as solid (—) lines, while the defective pathway as dashed (----) lines. Arrows indicate stimulatory effects, while blunt ends inhibitory effects.

Under normoglycemic conditions, acute elevation of extracellular glucose and intracellular cAMP levels stimulate NeuroD expression, whereas the same stimuli are interpreted as repressive signals under hyperglycemic conditions (Figure 1E and 2C). This divergent response is determined by the tonic level of pCREB and the continuance of ICER expression. In the absence of a deactivation pathway operated by PP2A under chronic hyperglycemia, CREB remains constantly activated, and further stimulation by glucose or forskolin above a physiological threshold prolongs the expression of a negative regulator ICER. Excessive production of ICER proteins in turn leads to repression of the NeuroD promoter. As insulin transcription is negatively regulated by ICER [25] and positively by NeuroD [4] the insulin synthesis/expression can be influenced directly by ICER [25] and indirectly through ICER-mediated NeuroD silencing (Figure 8).

As mentioned above, NeuroD is essential for the normal development, functional maturation of pancreatic β-cells, and maintenance of pancreatic β-cell physiology [37], [38]. NeuroD regulates the expression of other factors critical in glucose sensing and insulin exocytosis. These proteins include glucokinase (hexokinase IV), a rate-limiting enzyme of glucose metabolism [39]; the islet-specific glucose-6-phosphatase catalytic subunit-related protein [40]; secretin [41]; Pax6 [42]; SUR1, the regulatory subunit of the ATP-sensitive K^+^ channel [5]; SNAP25; syntaxin1A [43]; piccolo; and Noc2 [7]. Therefore, downregulation of NeuroD in chronic hyperglycemia may not only decrease insulin transcription but also attenuate insulin secretion [43] by affecting the glucose sensing and insulin exocytosis machinery. Taken together, ICER-mediated repression of NeuroD synthesis under hyperglycemic conditions may be a critical pathogenic process leading to the impaired insulin depletion, secretion (Figure 8).

In experiments performed under high glucose conditions, the CREs of ICER and NeuroD consistently responded to glucose or cAMP signals in the opposite ways such that ICER was upregulated while NeuroD was repressed both in islets and HIT cells (Figures 1–3). The opposite responses of CREs are possibly attributable to the several facts. First, the CRE of the ICER gene is activated by the active pCREB in the early phase after forskolin stimulus, whereas the CRE of NeuroD is influenced by the ratio of repressors to activators (ICER∶pCREB or ICER∶CREM) in the late phase [44]. The ICER∶CREM ratio is similarly shown to be a key regulator for homeostatic expression of pineal hormones such as melatonin in accordance with circadian rhythm in neuroendocrine cells [45], [46]. Second, diverse flanking sequences around CREs may display differential affinities for ICER, CREB, and CREM [25], [47]. Finally, the contribution of CREB signals to the NeuroD gene may be less than that of ICER, because NeuroD transcription is cooperatively regulated by several factors, including Ngn3 [34] and CREB (Figure 4), whereas ICER is solely regulated by CREB.

Ser/Thr protein phosphatases share substrates but differ in terms of metal ion requirement. Specifically, PP2A does not contain metal ions, whereas PP2B/calcineurin requires Ca^2+^ ions for optimal activity [48]. In the present study, we showed that PP2A is the key regulator that keeps the pCREB level below the physiological threshold under normoglycemic conditions. However, we cannot exclude the possibility that other Ser/Thr phosphatases, such as PP1 and PP2B/calcineurin, also play roles in pancreatic islets or β-cells. Indeed, several lines of evidence suggest associations of PP1, PP2A, and PP2B/calcineurin expression levels with diabetes in human patients. As mentioned above, earlier ethnographic studies have found that defective PP1 is a risk factor for non-insulin dependent diabetes mellitus (NIDDM) in Pima Indians of the southwest United States [18]. Clinical studies further have revealed that both PP1 and PP2A are decreased in the skeletal muscle of patients with NIDDM [15]–[17]. Depletion of the PP2A catalytic subunit markedly attenuates glucose-stimulated insulin secretion from pancreatic β-cells [20]. As PP2A knockout is embryonically lethal in mice [23], the role of PP2A in pathogenesis of diabetes remains to be determined. Interestingly, transgenic mice harboring a defective PP2A gene has been utilized as a model of Alzheimer's disease [49], suggesting that a common pathway is operative in neurons and pancreatic β-cells (*see below*). Inhibitors of PP2B/calcineurin, including FK506 and cyclosporin A, suppressed insulin mRNA level as well as insulin secretion in isolated pancreatic islets and β-cell lines [50], [51]. Targeted deletion of PP2B in pancreatic β-cells induces age-dependent diabetes characterized by decreased β-cell proliferation and reduced β-cell mass with concomitant decreases in the levels of β-cell transcription factors including MafA, Pdx-1, and NeuroD [21]. Paradoxically, transgenic mice overexpressing PP2B in β-cells also display diabetic symptoms with reduced β-cell mass [22]. Thus, fine control of Ser/Thr phosphatase levels may be crucial for maintenance of β-cell functions and mass [52]. Under our experimental conditions, the effect of PP1 and PP2B was minor compared to that of PP2A in HIT cells (Figure 6), possibly because of variations in the *in vitro* culture conditions used to grow/maintain pancreatic β-cells and insulinoma cells.

CREB functions as a key intersection point, at which the metabolic signals of circulating glucose level, and hormonal cues meet. Glucose uptake increases the cellular ATP∶ADP ratio, and eventually leads to increased intracellular Ca^2+^ concentration via closure of ATP-sensitive K^+^ channels and opening of voltage-gated L-type Ca^2+^ channels [53]. Ca^2+^ influx initiated by glucose stimulation activates Ca^2+^-calmodulin-dependent protein kinase, whereas cAMP signals triggered by hormonal cues or forskolin activate cAMP-dependent proteinase A (PKA). These Ser/Thr kinases phosphorylate CREB at Ser133 and promote transactivatory potential [10]. In the present study, acute challenges with 15 mM glucose or 30 µM forskolin are equally effective in prolonging ICER induction in pancreatic islet cells. However, the differential effects of glucose and forskolin on insulin secretion is also possible since insulin secretion is a complex consequence resulted from glucose transport/sensing, insulin synthesis/expression, membrane depolarization, and insulin exocytosis [54]–[56]. The differential effects of glucose and forskolin on insulin secretion maybe partially ascribed to the fact that glucose influences all these processes while forskolin mainly regulates the insulin expression with CREB-dependent pathways [25], [57]. Recent studies have shown that although CREB activity is potentiated by PKA, CREB phosphorylation alone is not a reliable predictor of target gene activation, and additional CREB regulatory partners such as such CREB binding protein (CBP) and cAMP-regulated transcriptional co-activator2 (CRTC2) are required for recruitment of the transcriptional apparatus to the promoter [10], [58]. Thus, multiple regulatory pathways triggered by circulating glucose and hormonal cues converge on the CREB activity in pancreatic β-cells via CREB phosphorylation and dephosphorylation at Ser133, CBP, and CRTC2 binding, and competition with ICER.

ICER has been identified in a variety of brain regions, including the cerebral cortex, the hippocampus, hypothalamic nuclei, dentate gyrus, and cerebellum [59] where NeuroD is also expressed [60], [61]. Notably, NeuroD contributes to the development of neuronal cells [38], exocytotic processes [43], and synaptic maturation [62]. Therefore, constant silencing of NeuroD by ICER may facilitate progression of the neuropathological defects that occur as diabetic complications. Interestingly, diabetes mellitus and reduced PP2A activity have been implicated as risk factors for Alzheimer's disease, although the molecular links are yet to be fully clarified [63]–[65]. Given that NeuroD and ICER play essential roles in neurons and neuroendocrine cells, the present study provides novel insights into the potential common processes by which chronic hyperglycemia accompanies not only β-cell dysfunction but also diabetic neuropathy.

In conclusion, a decrease in Ser/Thr phosphatase activity under conditions of chronic hyperglycemia is one of important process for gradual depletion of insulin reservoir during the progression of β-cell dysfunction. Interruption of the vicious cycle (Figure 8) triggered by the reduced phosphatase and the consequent ICER-mediated repression of NeuroD may be of clinical value in preventing β-cell failure and glucotoxicity in patients with Type 2 diabetes.

## Materials and Methods

### Ethics Statements

The Animal Care Committee of the Catholic University of South Korea approved the experimental protocol by the Institutional Animal and Use Committee (IACUC No. 2009-0093-04), and all procedures performed in this study were followed by ethical guidelines for animal studies.

### Plasmids

The reporter plasmids used in this study containing the proximal promoter regions of NeuroD, including pGL3-NeuroD(−2.2 kb) and pGL3-NeuroD(−100 bp) have been described previously by Huang *et al.*, 2000 [34]; an CREB expression vector by Ghil *et al.*, 2000 [66]; expression vectors for CREMτα and ICER Iγ by Inada *et al.*, 1999 [25]; the catalytic subunit of PP2B-Aα/calcinurin by Oliveria *et al.*, 2003 [67]. Expression vectors for PP1 and PP2A were generated by inserting the coding region of each catalytic subunit into the pLenti M1.4-MCMV (Macrogen, Seoul, South Korea) ; PP2A Cα (gi: 13277983) with BamHI and ClaI, PP1α (gi: 133892519) with XbaI and ClaI. The CRE site within the 1,025 bp fragment of the NeuroD promoter was mutagenized through multiple steps of PCR using Taq polymerase (Roche, Mannheim, Germany) and finally inserted to pGL3-NeuroD(−2.2 kb) that was digested with NheI and XhoI. The 224 bp fragment with mCRE was inserted into Kpn1 and Nco1 site to replace the wild type CRE in pGL3-NeuroD(−100 bp).

### Pancreatic islet cells

Rat pancreatic islets were isolated from Sprague-Dawley rats (200–230 g) by digesting the pancreatic duct with 1 mg/ml collagenase P (Roche) in phosphate buffered saline (PBS) and separated with Histopaque-1077 (Sigma, St. Louis, MO) as previously described [68]. The islets with similar sizes (100–150 µm) were hand-picked under a dissecting microscope and incubated in PRMI 1640 (Invitrogen, Carlsbad, CA) containing 1,800 mg/l (10 mM) glucose, 10% FBS, 100 unit/ml penicillin, 100 µg/ml streptomycine in uncoated petridish for 24 h. Then islets were divided into two groups and preconditioned for 8 days either in RPMI 1640 with 10% fetal bovine serum (FBS) containing 900 mg/l glucose (5 mM, low/normal level) or 5400 mg/l glucose (30 mM, high level) at 37°C under 5% CO_2_ atmosphere. During this period, medium was replaced with fresh one every other day. Before RT-PCR analysis, the islet cells from each group were pre-conditioned in fresh RPMI with 10% FBS containing 900 mg/l (5 mM) glucose for 2 h at 37°C and then transferred to fresh RPMI 1640 containing 15 mM glucose (Figure 1A). After incubation for the indicated period of time, total RNA was isolated and subject to SYBR green real-time or semi-quantitative RT-PCR. Alternatively, after pre-incubation in the presence of 5 mM glucose for 2 h, the islet cells were transferred to fresh RPMI 1640 containing 5 mM or 30 mM glucose and then, forskolin (Sigma) was added to a final concentration of 30 µM (Figure 2A) to avoid the pleiotropic effects of GLP-1 on β-cells [69] and directly activate adenylyl cyclase to increase the intracellular levels of cAMP. As shown in Figure 3 and Supporting Figure S4, both 30 µM forskolin and 10 µM forskolin persistently induced the ICER expression in HIT cells after long-term cultivation in the presence of 25 mM glucose. The similar results with 10 µM and 30 µM forskolin suggest that our original data obtained with 30 µM forskolin are reliable. Therefore, we utilized 30 µM forskolin throughout our experiments. The homogeneity and the viability of islets were verified with dithizone (DTZ) staining in the presence of 5 mM or 30 mM glucose (Figure 1B). Briefly, pancreatic islets were incubated in RPMI 1640 medium containing 0.1 mg/ml DTZ (Sigma) for 5 min at room temperature. Islets were stained crimson red to determine homogeneity and viability of islets.

### Insulin secretion and insulin content assay

Isolated rat islets incubated under low glucose (5 mM) and hyperglycemic (30 mM) conditions for 8 days were washed in KRB washing buffer (130 mM NaCl, 3.6 mM KCl, 1.5 mM CaCl_2_, 0.5 mM MgSO_4_, 0.5 mM KH_2_PO_4_, 2.0 mM NaHCO_3_, and 10 mM Hepes, pH 7.4) and incubated in KRB buffer containing 5 mM glucose at 37°C for 2 h. Then, 30 islets with similar sizes were stimulated with 15 mM glucose in 1 ml KRB buffer at 37°C and the KRB buffer was collected 6 h later for quantification of secreted insulin with radioimmunoassay (RIA) kit (Linco, St. Charles, MO). The islets were harvested and total intracellular insulin content were extracted by incubating them overnight in 1% hydrochloric acid (ethanol/H_2_O_2_/HCl, 14∶57∶3) at 4°C. After sonication and centrifugation, 200 µl of the supernatant was used for RIA to determine the intracellular insulin content and 800 µl for Bradford assay to determine the total proteins.

### HIT-T15 cell culture

HIT-T15 cells (hamster insulinoma tumor cells) from American Type Culture Collection (Cat. No. CRL-1777, ATCC, Manassas, VA 20108,) were grown in DMEM (Invitrogen) with 10% FBS containing 990 mg/l (5.5 mM, low/normal level) or 4,500 mg/l (25 mM, high level) glucose for at least two weeks. To stably overexpress PP2A Cα, HIT cells were transfected with a PP2A Cα expression vector and selected in the presence of 10 µg/ml puromycin (Sigma) for 2 weeks.

### Reporter gene assay

One day prior to transfection, HIT cells were plated at a density of 3×10^5^ cells per well in 6-well plates. Transfection was carried out with indicated amount of reporter plasmids and expression vectors using Lipofectamine Plus (Invitrogen), following the manufacturer's recommendations. The day after transfection, the medium was replaced with fresh growth medium containing 0.5% FBS, and cell growth continued for 24 h. Forty hours after transfection, forskolin was added at a final concentration of 30 µM for the indicated time-period before harvesting cells. Luciferase activity was determined with cell extracts using the Dual-Luciferase assay system (Promega, Madison, WI). During transfection, the total DNA amount was kept constant by adding pcDNA3 (Invitrogen). A plasmid for Renilla LUC-thymidine kinase (Promega) was used as an internal control to normalize transfection efficiency. Normalized luciferase activity was presented as a fold ratio in relation to the basal activity of the reporter gene in the absence of expression vectors or forskolin.

### Quantitative real-time PCR and semi-quantitative RT-PCR analysis

Isolated rat islets or HIT cells were incubated under low glucose and high glucose conditions as mentioned above. Then, total RNA was isolated from cells using RNAzol B (Tel-Test, Friendswood, TX), and cDNA synthesized from 1 µg of RNA using the First-strand cDNA synthesis kit (Roche). Two µl of reaction product from reverse transcription for first-strand cDNA was used for SYBR green quantitative real-time PCR for islets using a Rotor-gene Q, Qiagen Thermal-cycler equipment (Qiagen, Valencia, CA) and power SYBR® Green PCR master mix (Applied Biosystems, Foster city, CA). Primers for NeuroD, ICER I, SUR1, Insulin I/II in SYBR green real-time PCR were designed to recognize the separate exons of both rat and hamster genes to exclude the possibility of amplifying genomic DNA (Supporting Table S1). The PCR conditions for each set of primers were as follows: after initial denaturation at 95°C for 10 min, 40 cycles of denaturation at 95°C for 15 sec, annealing at indicated temperature for 30 sec, and extension at 72°C for 40 sec (Supporting Table S1). Following the final amplification cycle, a melting curve was acquired by one cycle of heating at 72°C for 1 min, and then increasing the temperature to 95°C at a rate of 1°C per min. The specificity of PCR reaction was ensured by single peak in melting curve analysis. The original values of cycle threshold (Ct) are described in Supporting Table S3. The results were normalized to the expression levels of the GAPDH reference gene and the relative mRNA levels were calculated using the 2^−ΔΔCt^ method [70]. The data represent means ± S.E. of three independent experiments, expressed as fold ratio of mRNA expression compared with the control value (islets cultivated in 5 mM glucose without 15 mM glucose stimulus in Figure 1 or 30 µM forskolin treatment in Figures 2). Each experiment was carried out in duplicates. Traditional, semi-quantitative RT-PCR was also carried out with the same sets of primers for NeuroD, SUR1, insulin, pdx-1, PP1α, PP2B-Aα, and PP2A Cα and GAPDH except ICER (Supporting Table S1). The ICER-specific primer set could detect two splice variants, ICER I and ICER Iγ. PCR was performed with an initial denaturation at 98°C for 1 min; 30 cycles for each genes comprising 95°C for 1 min, annealing at the indicated temperature for 30 sec, and extension at 72°C for 1 min; and a final extension at 72°C for 7 min. PCR products were separated by electrophoresis on 2% agarose gels. Signals from traditional RT-PCR results were captured with a VersaDoc 4000 Imager (Bio-Rad Laboratories, Hercules, CA) and normalized to the value of GAPDH. PCR with 28∼32 cycles yielded similar extent of amplification (Supporting Figure S1), indicating the PCR reaction was not saturated. The results were also similar to those obtained with SYBR green real-time PCR (compare Figure 1D–H with Supporting Figure S1), suggesting the reliability of our traditional, semi-quantitative RT-PCR data performed with HIT cells.

### Western blot analysis

Cells were lysed in RIPA buffer (50 mM Tris; pH 7.4, 1 M NaCl, 1% NP-40, 1% sodium deoxycholate, 0.1% sodium dodecyl sulphate) using a standard protocol and subjectect to polyacryl amide gel electrophoresis. After transferred to PVDF membrane, the protein was identified with the primary antibodies: phospho-CREB (1∶1000, polyclonal, Cell Signaling, Danvers, MA), catalytic subunits of PP1α, PP2B-Aα, and PP2A Cα (1∶1000, monoclonal, Upstate, Temecula, CA). Immunoreactivity was visualized using horse radish peroxidase-conjugated anti-rabbit or mouse IgG antibodies (1∶5000, Zymed, San Francisco, CA) and the SuperSignal Chemiluminescence Substrate kit (PIERCE, Rockford, IL). To confirm equal loading, blots were stripped and reprobed with antibodies against CREB (1∶1000, Cell Signaling) or α-actin (1∶1000, Sigma).

### Chromatin immunoprecipitation (ChIP) assay

HIT cells (1.5×10^6^/100 mm dish) were incubated in 1% (v/v) formaldehyde, lysed, and subjected to immunoprecipitation with 1 µg of anti-ICER antibody (SantaCruz Biotechnology Inc., Santa Cruz, CA). ICER-bound DNA was precipitated using a ChIP assay kit (Upstate), following the manufacturer's recommendation, and resuspended in 20 µl of H_2_O. Amplification was conducted with 2 µl DNA solution under the following conditions: initial denaturation at 98°C for 1 min, 35 cycles of 94°C for 1 min, 52°C for 40 sec, and 72°C for 1 min, and a final extension step at 72°C for 7 min. The PCR primers designed for amplifying the proximal NeuroD promoter were 5′-aaagttctggggaggggtgaatgag-3′ (−160/−134; forward) and 5′-cttgccttctgcgtgggcgaattcc-3′ (+5/+29 bp; reverse). The 189 bp PCR product was analyzed on a 2% agarose gel.

### PP2A activity assay

HIT cells (3×10^5^/35 mm dish) were harvested in phosphatase extraction buffer (100 mM Tris-HCI, pH 7.6, 2 mM MgCl_2_, 1 mM CaCl_2_, 2 mM EGTA, 2 mM EDTA, 1% Triton X-100, 1 mM PMSF, protease inhibitor cocktail). After sonification, the clear lysates were precleared with 50% (w/v) protein A Sepharose bead (Amersham, Little Chalfont, Buckinghamshire, UK) and then incubated with 4 µg of antibody against the catalytic subunit of PP2A Cα (Upstate). After washing with PBS, the remaining activity in immunoprecipitates was assessed using a Ser/Thr phosphatase assay kit (Upstate) with 0.75 mM synthetic PP2A-specific phosphopeptide (K-R-pT-I-R-R) as a substrate for 10 min at 30°C. Free phosphate in a 25 µl reaction volume was colorimetrically measured at 650 nm after reaction with malachite green as recommended by the manufacturer. Specific activity of phosphatase (pmoles of phosphate/min/µg protein) was determined by comparing with controls containing no enzyme or a freshly prepared phosphate standard. The data from three independent experiments are presented as the mean ± S.E. with respect to the value in the presence of 5 mM glucose.

### Preperation of PP2A siRNA and transfection

HIT cells were transfected with 50 nM siRNA for silencing PP2A Cα (5′-tggaacttgacgacactcttaa-3′) or scrambled siRNA (Genolution, Seoul, South Korea), using Lipofectamine RNAi MAX (Invitrogen). The following day, the medium was replaced with fresh DMEM (5.5 mM glucose) containing 0.5% FBS, and cell growth continued for 24 h. Silencing or overexpression of PP2A was verified by RT-PCR and western analysis (Figure 7A).

### Statistical analysis

Signals from western blot, RT-PCR and ChIP analysis were captured with a VersaDoc 4000 Imager (Bio-Rad Laboratories). All experiments were conducted a minimum of 3 times, and data presented as means ± S.E. *P* values for statistical significance were estimated with respect to the indicated control value using the *t* test (*^/#^, *P*<0.05; **^/##^, *P*<0.01).

## Supporting Information

Figure S1 **The effects of chronic hyperglycemia on β-cell specific genes in rat islets were analyzed using semi-quantitative RT-PCR.** (**A**) The mRNA levels of indicated genes in various conditions as shown in Figure 1A were also analyzed using traditional, semi-quantitative RT-PCR analyses. Two isoforms of ICER I (168 bp) and its splice variant, ICER Iγ (129 bp) were identified with the same set of primers, while non-allelic Ins 1 and Ins 2 were detected as s single band (Supporting Table S1). The effects of chronic hyperglycemia on gene expression were similar when PCR was carried out for 28∼32 cycles, suggesting that there was no saturation of the PCR amplification or ethidium-bromide staining. (**B**) RT-PCR results from amplification for 30 cycles were semi-quantitatively measured and normalized to that of GAPDH. Data from three independent experiments are presented as average fold ratios with respect to the value of 5 mM glucose-cultured islets before glucose stimulation. The overall effects of chronic hyperglycemia on gene expression were similar to the results obtained with SYBR green real-time PCR as shown in Figure 1, verifying that the semi-quantitative RT-PCR data in the subsequent studies with HIT cells were also reliable to demonstrate the relative mRNA level. Significant effects of 15 mM glucose (*, *P*<0.05; **, *P*<0.01) or 8-day incubation in 30 mM glucose (*^#^*, *P*<0.05) were marked.
(TIF)

Figure S2 **The effects of chronic hyperglycemia on the responsiveness to forskolin in rat islets were analyzed using semi-quantitative RT-PCR.** (**A**) Semi-quantitative RT-PCR from islet cells cultured in various conditions as shown in Figure 2A also represents the chronic effect of hyperglycemia on the expression of ICER, NeuroD, SUR1, and insulin gene. (**B**) RT-PCR results from amplification for 30 cycles were semi-quantitatively measured and normalized to that of GAPDH. Data from three independent experiments are presented as average fold ratios with respect to the value of 5 mM glucose-cultured islets prior to addition of forskolin. The overall effects of chronic hyperglycemia on the responsiveness to forskolin were similar to the results obtained with SYBR green real-time PCR as shown in Figure 2, verifying that the semi-quantitative RT-PCR data from HIT cells in the present study were also reliable to demonstrate the relative mRNA level. Significant effects of forskolin (*, *P*<0.05; **, *P*<0.01) or 8-day incubation in 30 mM glucose (*^#^*, *P*<0.05) were marked.
(TIF)

Figure S3 **Cloning of hamster PP2A Cα.** The hamster PP2A Cα cDNA was cloned (gi:325504919) in this study. The sequence analysis indicated 97.5% identity at the DNA level and 99.97% identity at the protein level among the mammals. Nucleotide sequences used for siRNA are marked with the solid-line and the primers used for RT-PCR analysis are marked with dotted lines.
(TIF)

Figure S4 **To validate the effectiveness of 30 µM forskolin.** To avoid the pleiotropic effects of GLP-1 on β-cells and directly activate adenylyl cyclase to increase the intracellular levels of cAMP, we utilized 30 µM forskolin throughout our experiments. As shown in Figure 3 with 30 µM forskolin, 10 µM forskolin also persistently induced the ICER expression in HIT cells after long-term cultivation in the presence of 25 mM glucose. The similar results with 10 µM and 30 µM forskolin suggest that our original data obtained with 30 µM forskolin are reliable.
(TIF)

Table S1 **Experimental conditions for SYBR green real-time PCR and traditional RT-PCR.** Both rat (r) and hamster (h) gene specific primers for NeuroD, ICER I, SUR1, Insulin I/II, PP1α, CaN, and PP2A Cα were designed to recognize the separate exons to exclude the possibility of amplifying contaminating genomic DNA. Blast analysis showed high sequence homology for each gene between rodents and mammals ranging from 92 to 98%. The GenBank numbers for rat and hamster sequences were used to design the common primers for semi-quantitative RT-PCR and SYBR green real-time PCR except for the ICER gene. The ICER-specific primers recognized two isoforms, ICER I (168 bp), and its splice variant, ICER Iγ (129 bp) in traditional, semi-quantitative RT-PCR. However, this primer set interfered with real-time amplification, thus we designed a new primer for SYBR green real-time PCR which only recognized ICER I but not lacking domain called γ in ICER Iγ. For the insulin genes, two nonallelic genes (insulin I and II) displaying more than 90% homology [71], [72] were detected as a 308 bp product with the same set of primers.
(TIF)

Table S2 **CRE-like sequences are conserved at the proximal promoter region of NeuroD.** In the mouse NeuroD gene, the CRE like sequence is found −73 bp from transcription initiation site, which is 43 bp upstream of the TATA box. The CRE-like sequence (TCAGCT^C^/_A_^A^/_G_) is highly conserved among humans, primates and rodents, suggesting functional significance through evolution.
(TIF)

Table S3 **Ct mean values from SYBR green real-time RT-PCR of β-cell specific genes expressed in pancreatic rat islets.** Rat pancreatic islet cells were cultured in the presence of 5 mM or 30 mM glucose for 8 days before being challenged with 15 mM glucose or 30 µM forskolin (Figure 1A and 2A). Quantitative real time RT-PCR was carried out using SYBR green. Each experiment was carried out in duplicates and the mean values of cycle threshold (Ct) from three independent experiments were presented as means ± S.E. These Ct values were used to deduce relative mRNA levels using the 2^−ΔΔCt^ method. The relative mRNA level of each gene was normalized to the value of GAPDH and presented as a fold ratio with respect to the control value (non-glucose or forskolin treated-islet cells under conditions of 5 mM glucose) in Figure 1 and 2.
(TIF)
