# Supplementary figures and images for: Deregulation of CREB Signaling Pathway Induced by Chronic Hyperglycemia Downregulates NeuroD Transcription
Source: PLoS One. 2012 Apr 3;7(4):e34860. doi: 10.1371/journal.pone.0034860 (PMC3318007; doi:10.1371/journal.pone.0034860)

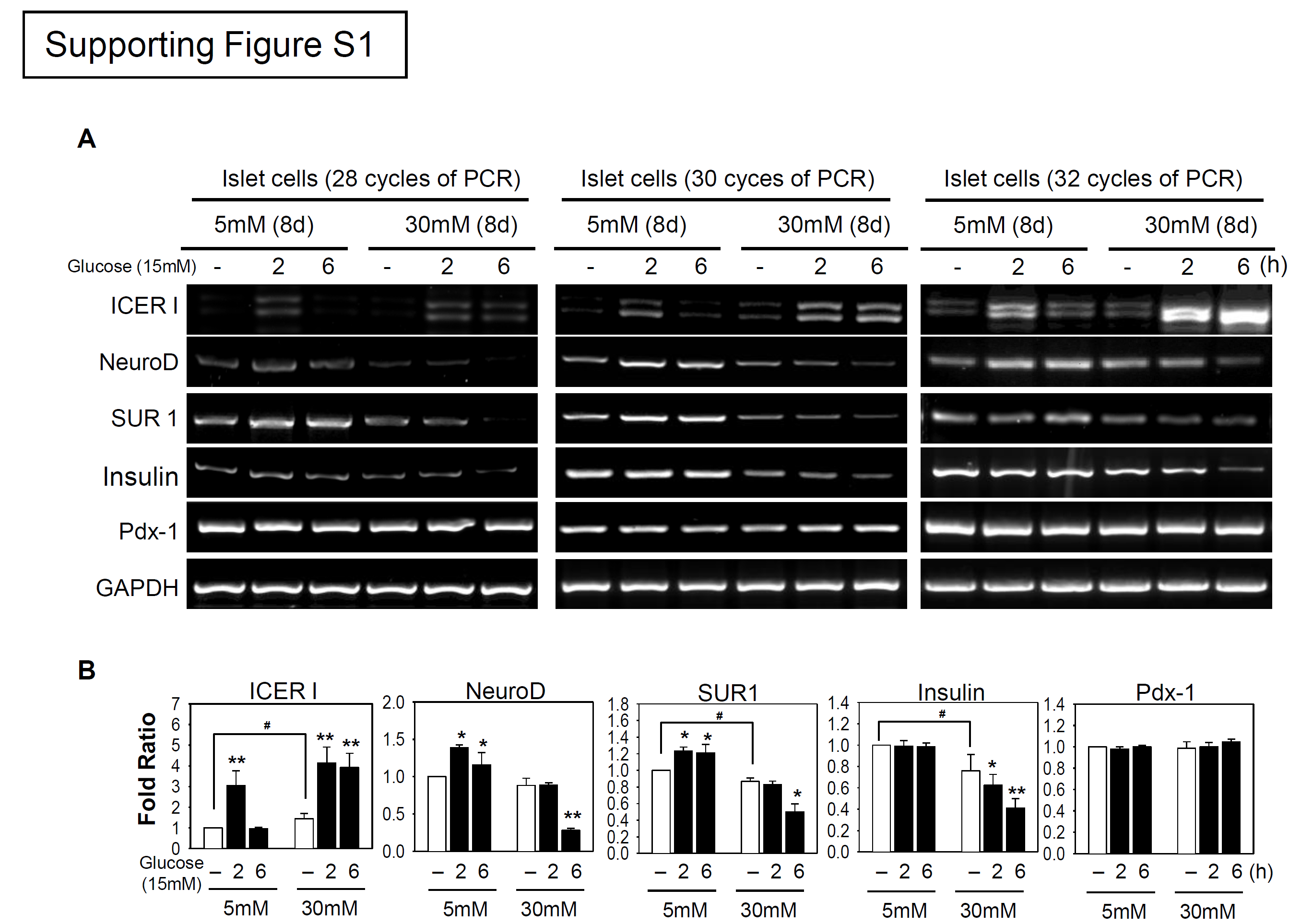

Supplement: Figure S1 — The effects of chronic hyperglycemia on β-cell specific genes in rat islets were analyzed using semi-quantitative RT-PCR. (A) The mRNA levels of indicated genes in various conditions as shown in Figure 1A were also analyzed using traditional, semi-quantitative RT-PCR analyses. Two isoforms of ICER I (168 bp) and its splice variant, ICER Iγ (129 bp) were identified with the same set of primers, while non-allelic Ins 1 and Ins 2 were detected as s single band (Supporting Table S1). The effects of chronic hyperglycemia on gene expression were similar when PCR was carried out for 28∼32 cycles, suggesting that there was no saturation of the PCR amplification or ethidium-bromide staining. (B) RT-PCR results from amplification for 30 cycles were semi-quantitatively measured and normalized to that of GAPDH. Data from three independent experiments are presented as average fold ratios with respect to the value of 5 mM glucose-cultured islets before glucose stimulation. The overall effects of chronic hyperglycemia on gene expression were similar to the results obtained with SYBR green real-time PCR as shown in Figure 1, verifying that the semi-quantitative RT-PCR data in the subsequent studies with HIT cells were also reliable to demonstrate the relative mRNA level. Significant effects of 15 mM glucose (*, P<0.05; **, P<0.01) or 8-day incubation in 30 mM glucose (#, P<0.05) were marked. (TIF) [file pone.0034860.s001.tif]

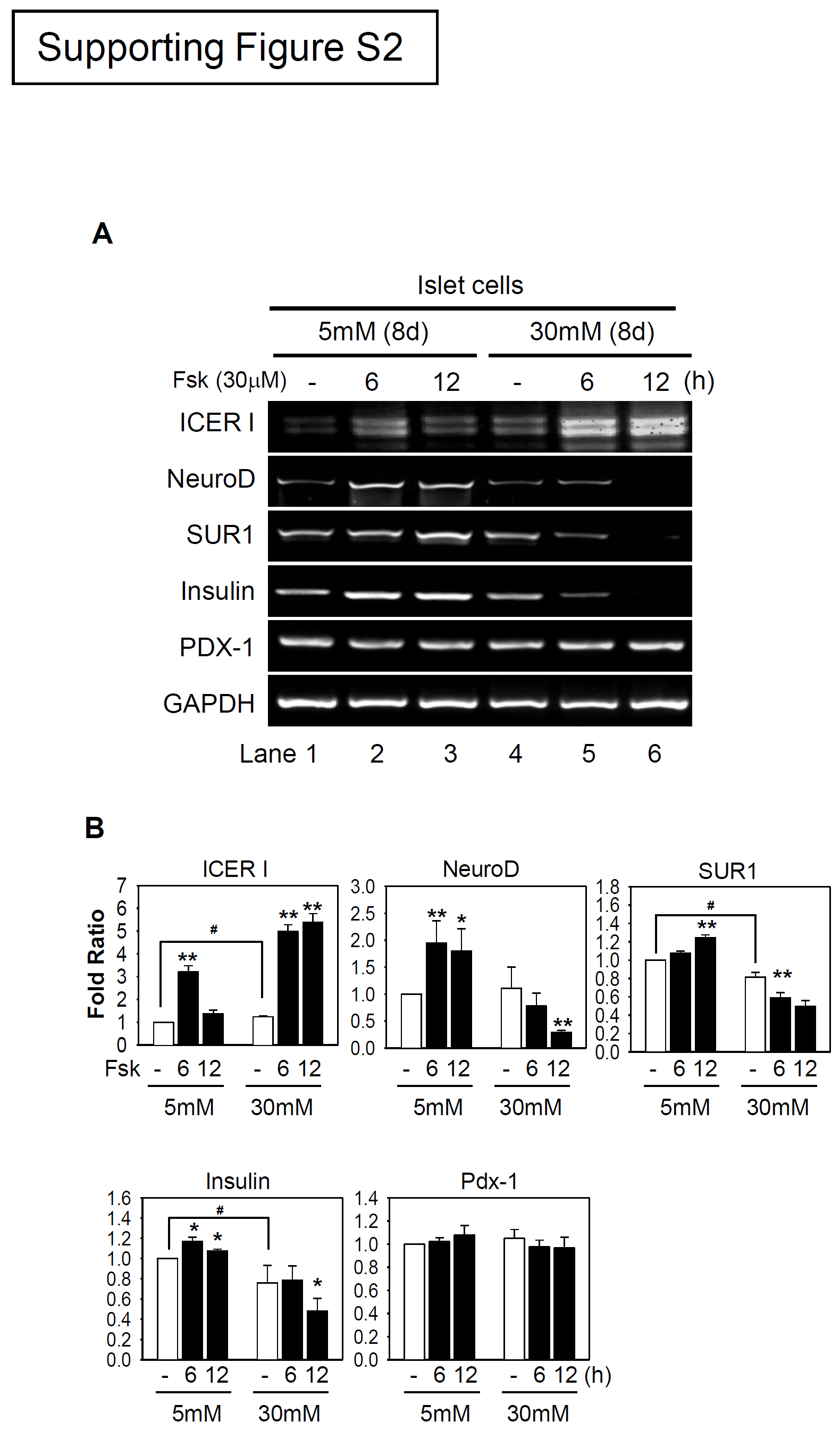

Supplement: Figure S2 — The effects of chronic hyperglycemia on the responsiveness to forskolin in rat islets were analyzed using semi-quantitative RT-PCR. (A) Semi-quantitative RT-PCR from islet cells cultured in various conditions as shown in Figure 2A also represents the chronic effect of hyperglycemia on the expression of ICER, NeuroD, SUR1, and insulin gene. (B) RT-PCR results from amplification for 30 cycles were semi-quantitatively measured and normalized to that of GAPDH. Data from three independent experiments are presented as average fold ratios with respect to the value of 5 mM glucose-cultured islets prior to addition of forskolin. The overall effects of chronic hyperglycemia on the responsiveness to forskolin were similar to the results obtained with SYBR green real-time PCR as shown in Figure 2, verifying that the semi-quantitative RT-PCR data from HIT cells in the present study were also reliable to demonstrate the relative mRNA level. Significant effects of forskolin (*, P<0.05; **, P<0.01) or 8-day incubation in 30 mM glucose (#, P<0.05) were marked. (TIF) [file pone.0034860.s002.tif]

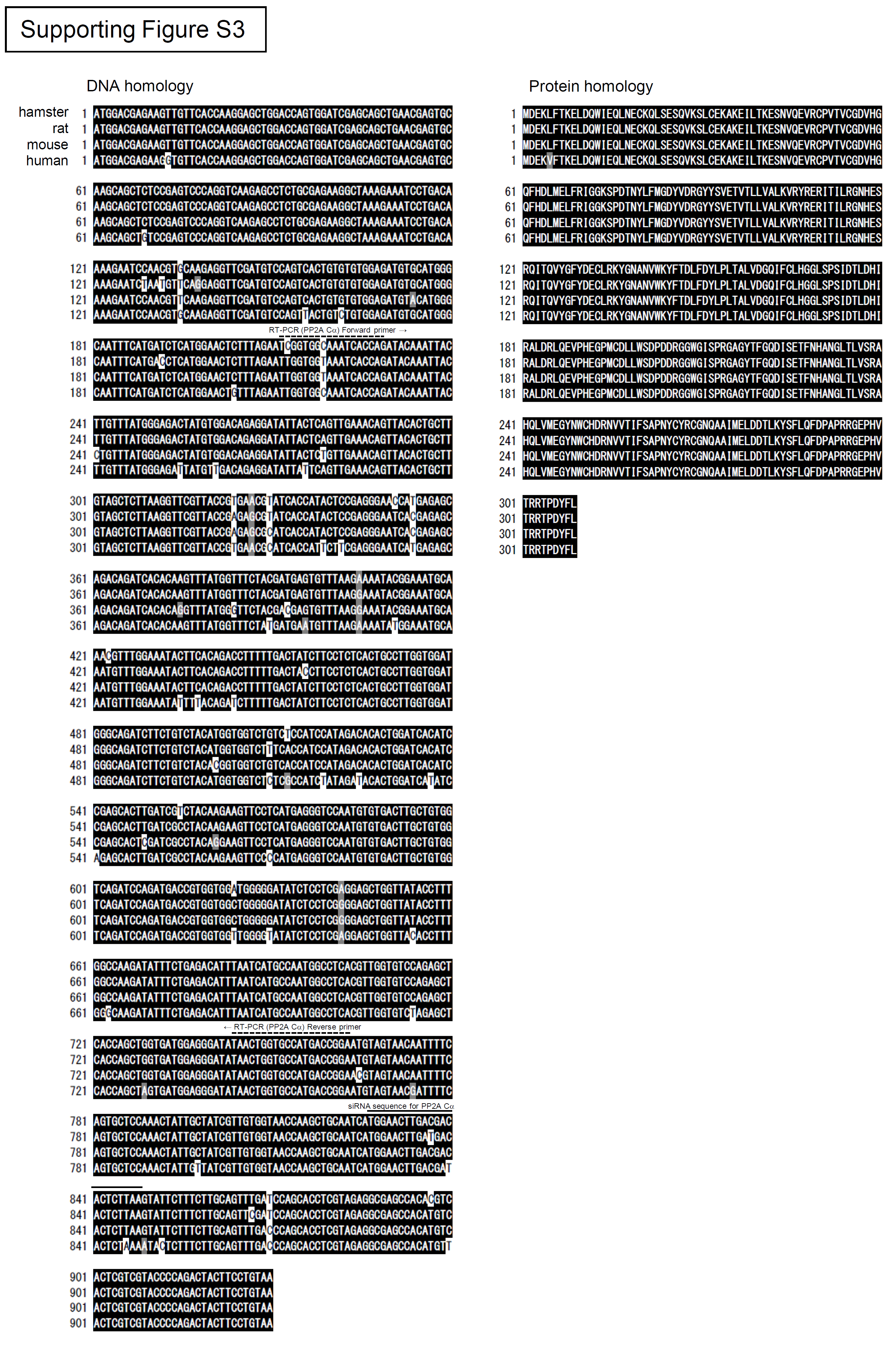

Supplement: Figure S3 — Cloning of hamster PP2A Cα. The hamster PP2A Cα cDNA was cloned (gi:325504919) in this study. The sequence analysis indicated 97.5% identity at the DNA level and 99.97% identity at the protein level among the mammals. Nucleotide sequences used for siRNA are marked with the solid-line and the primers used for RT-PCR analysis are marked with dotted lines. (TIF) [file pone.0034860.s003.tif]

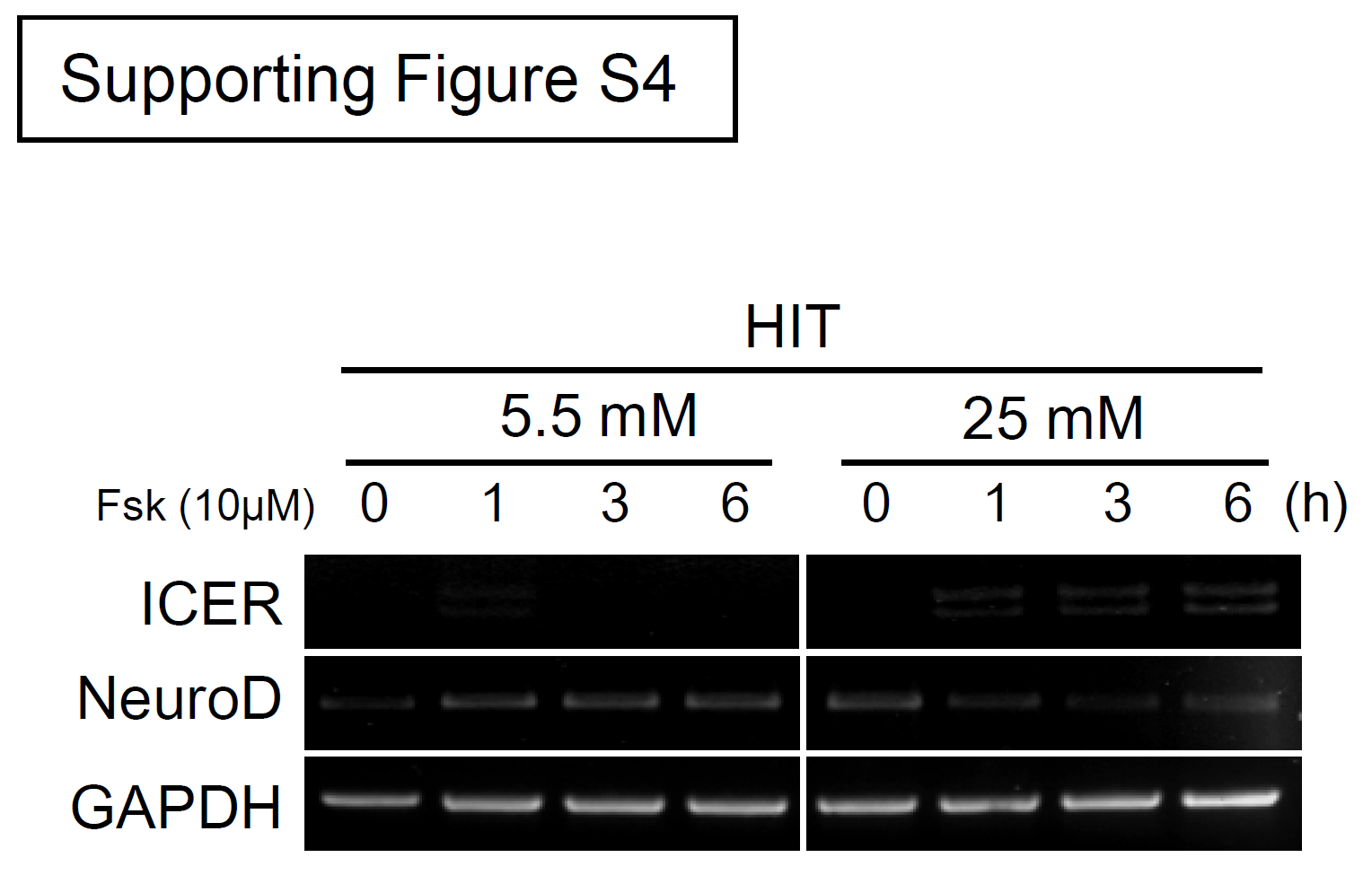

Supplement: Figure S4 — To validate the effectiveness of 30 µM forskolin. To avoid the pleiotropic effects of GLP-1 on β-cells and directly activate adenylyl cyclase to increase the intracellular levels of cAMP, we utilized 30 µM forskolin throughout our experiments. As shown in Figure 3 with 30 µM forskolin, 10 µM forskolin also persistently induced the ICER expression in HIT cells after long-term cultivation in the presence of 25 mM glucose. The similar results with 10 µM and 30 µM forskolin suggest that our original data obtained with 30 µM forskolin are reliable. (TIF) [file pone.0034860.s004.tif]

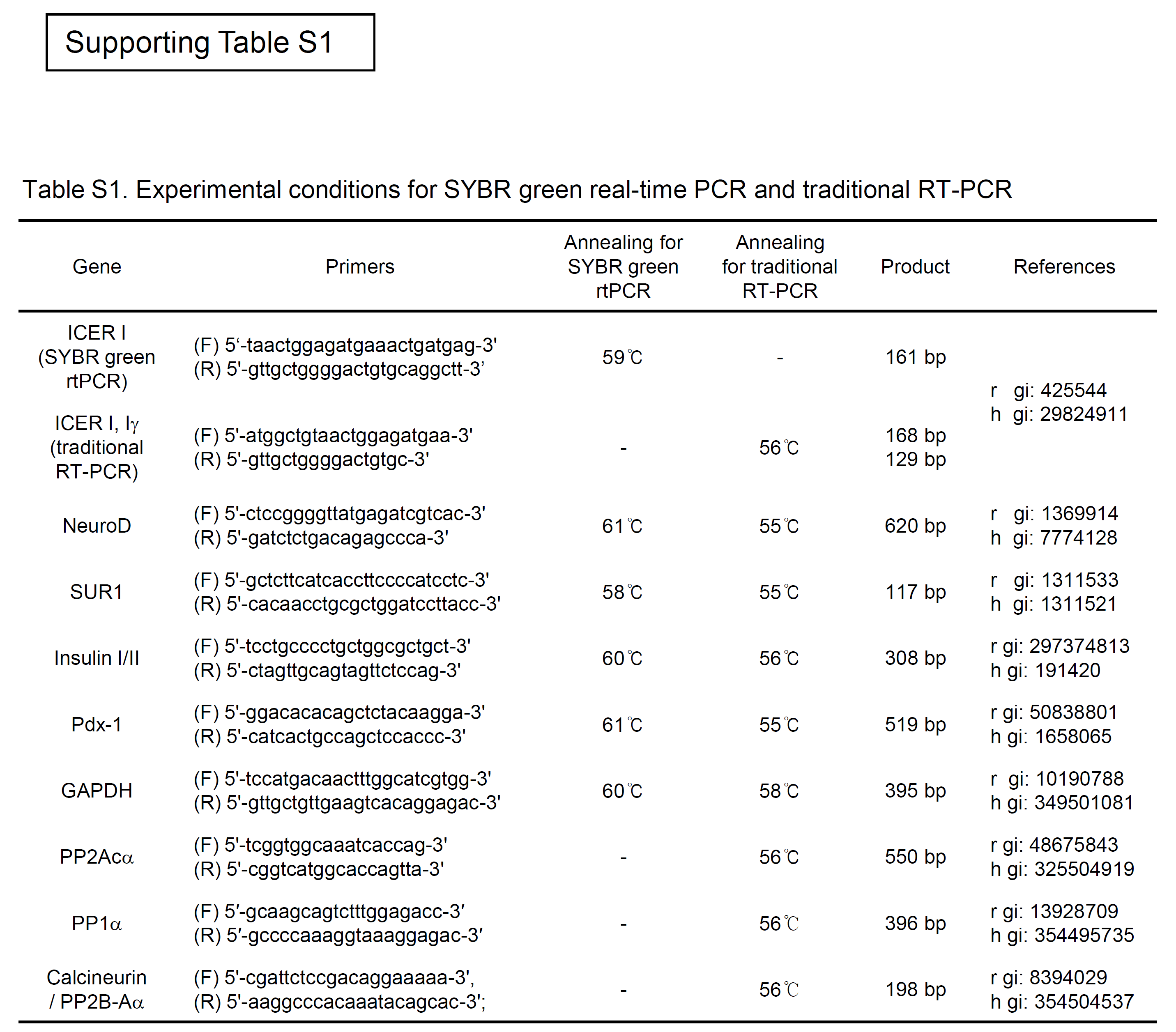

Supplement: Table S1 — Experimental conditions for SYBR green real-time PCR and traditional RT-PCR. Both rat (r) and hamster (h) gene specific primers for NeuroD, ICER I, SUR1, Insulin I/II, PP1α, CaN, and PP2A Cα were designed to recognize the separate exons to exclude the possibility of amplifying contaminating genomic DNA. Blast analysis showed high sequence homology for each gene between rodents and mammals ranging from 92 to 98%. The GenBank numbers for rat and hamster sequences were used to design the common primers for semi-quantitative RT-PCR and SYBR green real-time PCR except for the ICER gene. The ICER-specific primers recognized two isoforms, ICER I (168 bp), and its splice variant, ICER Iγ (129 bp) in traditional, semi-quantitative RT-PCR. However, this primer set interfered with real-time amplification, thus we designed a new primer for SYBR green real-time PCR which only recognized ICER I but not lacking domain called γ in ICER Iγ. For the insulin genes, two nonallelic genes (insulin I and II) displaying more than 90% homology [71], [72] were detected as a 308 bp product with the same set of primers. (TIF) [file pone.0034860.s005.tif]

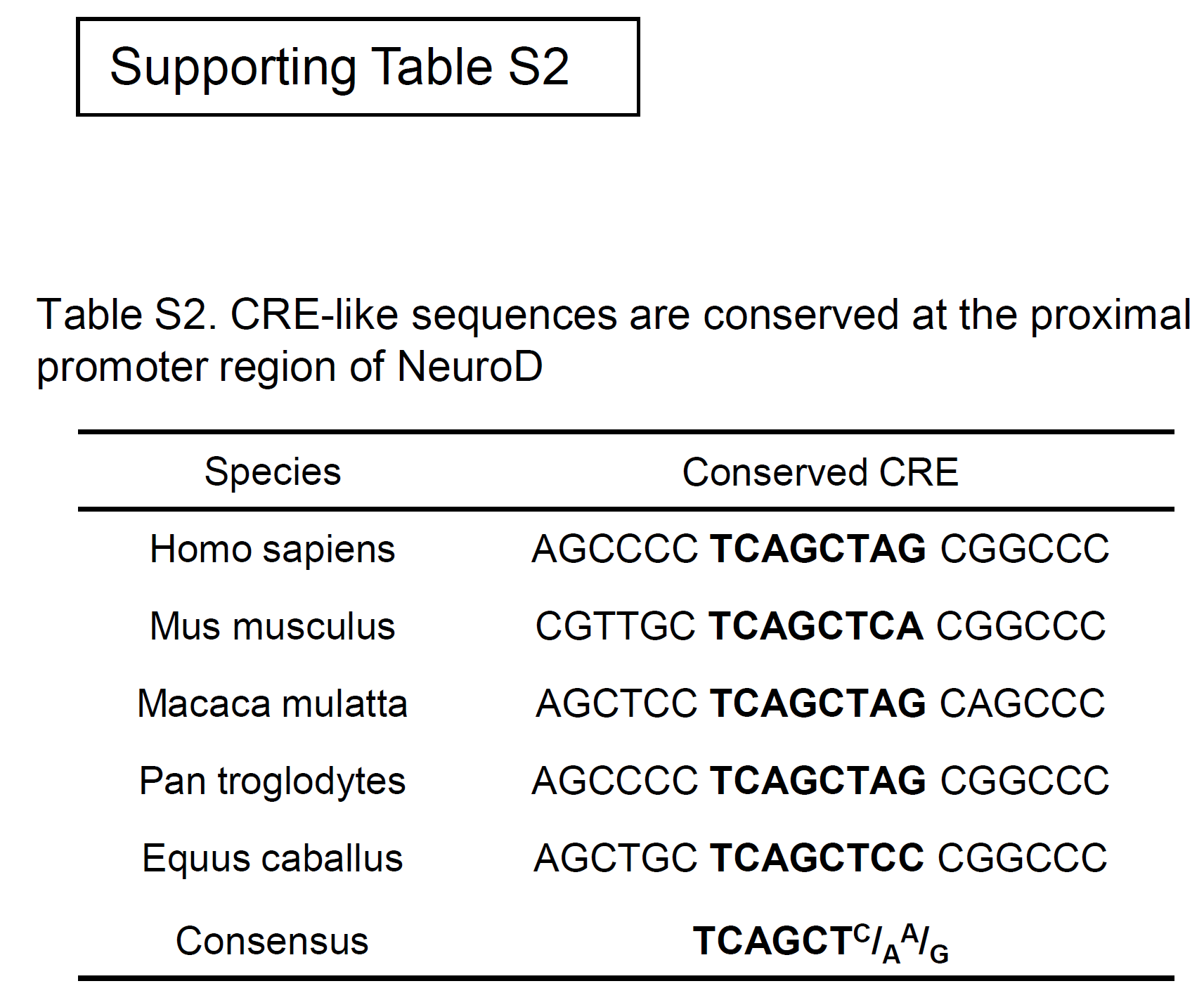

Supplement: Table S2 — CRE-like sequences are conserved at the proximal promoter region of NeuroD. In the mouse NeuroD gene, the CRE like sequence is found −73 bp from transcription initiation site, which is 43 bp upstream of the TATA box. The CRE-like sequence (TCAGCTC/A A/G) is highly conserved among humans, primates and rodents, suggesting functional significance through evolution. (TIF) [file pone.0034860.s006.tif]

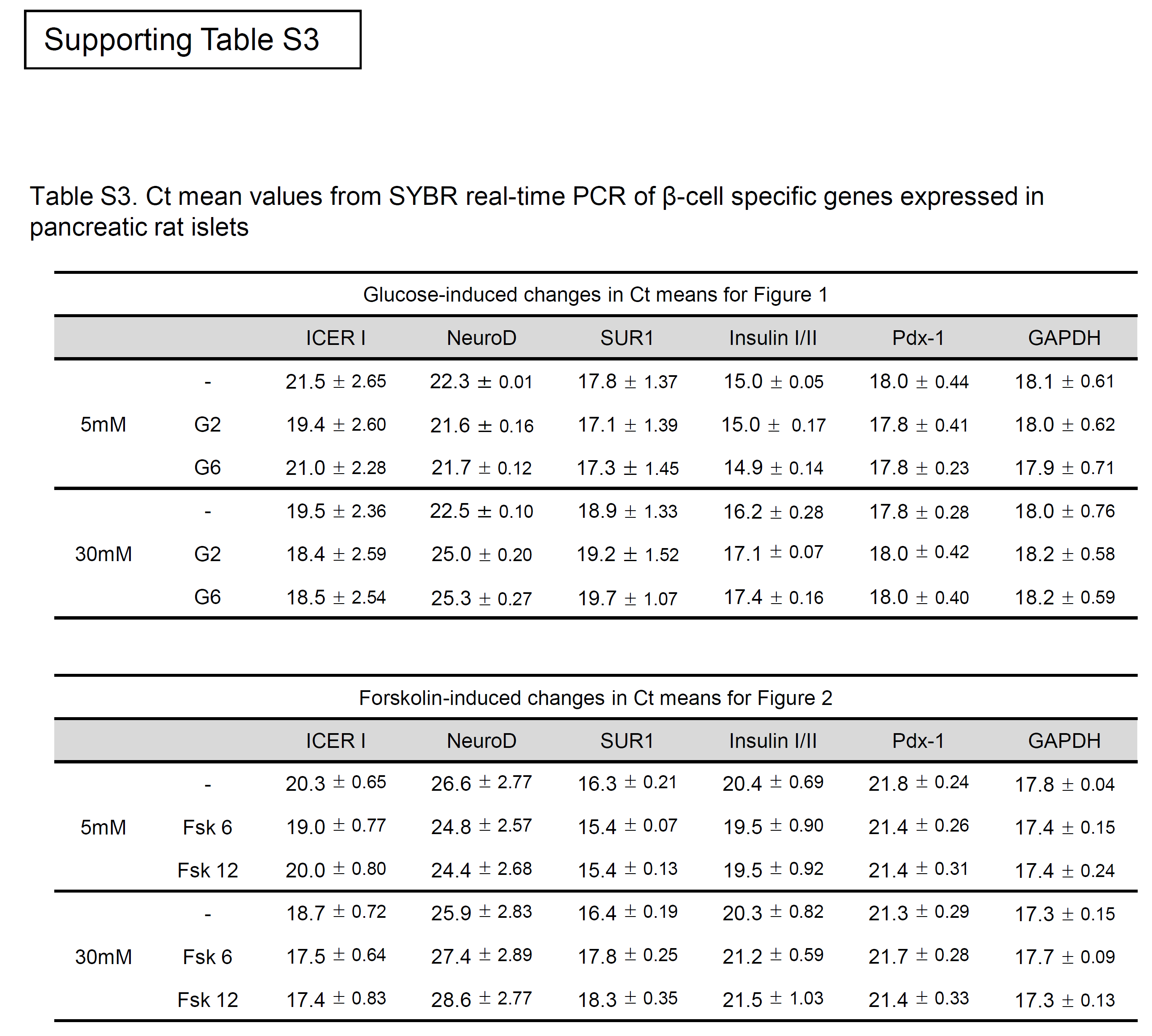

Supplement: Table S3 — Ct mean values from SYBR green real-time RT-PCR of β-cell specific genes expressed in pancreatic rat islets. Rat pancreatic islet cells were cultured in the presence of 5 mM or 30 mM glucose for 8 days before being challenged with 15 mM glucose or 30 µM forskolin (Figure 1A and 2A). Quantitative real time RT-PCR was carried out using SYBR green. Each experiment was carried out in duplicates and the mean values of cycle threshold (Ct) from three independent experiments were presented as means ± S.E. These Ct values were used to deduce relative mRNA levels using the 2−ΔΔCt method. The relative mRNA level of each gene was normalized to the value of GAPDH and presented as a fold ratio with respect to the control value (non-glucose or forskolin treated-islet cells under conditions of 5 mM glucose) in Figure 1 and 2. (TIF) [file pone.0034860.s007.tif]
